# Overexpression of MicroRNA *miR-30a* or *miR-191* in A549 Lung Cancer or BEAS-2B Normal Lung Cell Lines Does Not Alter Phenotype

**DOI:** 10.1371/journal.pone.0009219

**Published:** 2010-02-15

**Authors:** Santosh Kumar Patnaik, Eric Kannisto, Sai Yendamuri

**Affiliations:** 1 Department of Thoracic Surgery, Roswell Park Cancer Institute, Buffalo, New York, United States of America; 2 Department of Surgery, University at Buffalo, Buffalo, New York, United States of America; Roswell Park Cancer Institute, United States of America

## Abstract

**Background:**

MicroRNAs (miRNAs) are small, noncoding RNAs (ribonucleic acids) that regulate translation. Several miRNAs have been shown to be altered in whole cancer tissue compared to normal tissue when quantified by microarray. Based on previous such evidence of differential expression, we chose to study the functional significance of miRNAs *miR-30a* and *-191* alterations in human lung cancer.

**Methodology/Principal Findings:**

The functional significance of miRNAs *miR-30a* and *-191* was studied by creating stable transfectants of the lung adenocarcinoma cell line A549 and the immortalized bronchial epithelial cell line BEAS-2B with modest overexpression of *miR-30a* or *-191* using a lentiviral system. When compared to the corresponding controls, both cell lines overexpressing *miR-30a* or *-191* do not demonstrate any significant changes in cell cycle distribution, cell proliferation, adherent colony formation, soft agar colony formation, xenograft formation in a subcutaneous SCID mouse model, and drug sensitivity to doxorubicin and cisplatin. There is a modest increase in cell migration in cell lines overexpressing *miR-30a* compared to their controls.

**Conclusions/Significance:**

Overexpression of *miR-30a* or *-191* does not lead to an alteration in cell cycle, proliferation, xenograft formation, and chemosensitivity of A549 and BEAS-2B cell lines. Using microarray data from whole tumors to select specific miRNAs for functional study may be a suboptimal strategy.

## Introduction

The significance of non-coding RNAs in the regulation of cellular processes has come to be increasingly appreciated [1]. The best studied non-coding RNAs are microRNAs (miRNAs), 18–25 nt-long small RNA that epigenetically regulate translation by binding to a complementary “seed” sequence common to their “target” mRNA [2]. As this complementarity does not have to be perfect, a single miRNA can regulate the expression of several hundred genes simultaneously [3]. The explosion of microarray technology has led to its application in miRNA genomics. Therefore, before the function of a significant proportion of miRNAs were known, miRNA profiling of several normal and abnormal human tissues have been performed [4]. The distinct difference between the data obtained between miRNA profiling and mRNA profiling is in the magnitude of differential expression seen in the comparison groups. Whereas several fold changes are commonly seen in mRNA expression using these technologies, the fold changes with miRNA experiments are more modest and are typically in the 1- to 2-fold range. Therefore, experiments to study the biological relevance of these modest fold changes are important to evaluate the significance of these changes. Based on previously published miRNA microarray data demonstrating differences in expression in human cancers compared to the corresponding normal tissue, two miRNAs were chosen in this study for such biological experiments. These two miRNAs were chosen because of consistent alterations in their expression between normal and cancerous tissue of various organs, a bio-informatic analysis of their predicted targets that suggested important roles in cell proliferation and migration and little published work on their functional roles in lung cancer.

*MiR-30a* is located on human chromosome 6q.13 and is generated from an intronic transcriptional unit that produces three miRNAs: *miR-30a*, *-30c* and *-30e* [5]. Two mature forms of the gene exist – *miR-30a-3p* and *miR-30a-5p*. Several studies have detected changes in *miR-30a* expression in human cancer. Calin et al. demonstrated a down-regulation of *miR-30a* in chronic lymphocytic leukemia (CLL) patients [6]. Similarly, *miR-30a* is down-regulated in lung cancer [7], colon cancer [8], pancreatic cancer [9], metastatic hepatocellular cancer (compared to primary) [10] and in acute myeloid leukemia (AML) patients with a t(11q23) translocation (compared to other AML patients) [11]. *MiR-30a* is important for the development of the prefrontal cortex via the regulation of brain-derived neurotrophic factor (BDNF) [12] and in vertebrate hepatobiliary development [13]. Other experimentally confirmed targets of *miR-30a* are adenylate kinase (Ak1) and the GW182 protein (Tnrc6a), a component of the RNA interference silencing complex [13].

*MiR-191* is located on human chromosome 3p21.31 and is generated from an intronic transcriptional unit that can produce two miRNAs: *miR-191* and *-425* [5]. Only a single mature form of *miR-191* is known to exist. *MiR-191* expression is up-regulated in pancreatic [14], colon, lung and prostate [7] cancers. It is also up-regulated in AML patients with abnormal karyotypes [11], and down-regulated in patients with CLL [6]. *MiR-191* expression is also altered in lungs exposed to cigarette smoke [15]. No mRNA targets of *miR-191* have been experimentally verified.

In this study, we evaluate the phenotypic changes seen by constitutive over-expression of *miR-30a* and *-191*, on a lung adenocarcinoma cell line (A549) [16] and an immortalized bronchial epithelial cell line (BEAS-2B) [17]. The A549 cell line was chosen because of the existence of a large amount of experimental data on its use in various assays. The BEAS-2B cell line was chosen because it is the immortalized cell line closest to normal bronchial epithelium. As the effects of overexpression of a microRNA is context-specific, we compared the alterations in phenotype of malignant and non-malignant cells.

## Materials and Methods

### Ethics Statement

All in vivo studies were approved by the Institutional Animal Care and Use Committee of the Roswell Park Cancer Institute.

### Cell Culture

A549 human bronchioloalveolar lung carcinoma and BEAS-2B normal bronchial epithelial cell lines were obtained from ATCC® (Manassas, VA). Cells were cultured, respectively, in Dulbecco's modified Eagle medium (+DMEM; Invitrogen®, Carlsbad, CA) with 10% fetal bovine serum (PAA Laboratories®, Austria), and LHC-9 medium (Invitrogen®) at 37°C in 5% CO_2_. Stably-transfected cells were cultured in the presence of 2 µg/ml puromycin (Roche®, Indianapolis, IN).

### Generation of Stably Transfected Cell Lines

Single-stranded DNA oligonucleotides with human *pre-miR-30a* or *-191* (miRBase accession IDs MI0000088 and MI0000465, respectively) sequences and with restriction enzyme site overhangs were obtained from Integrated DNA Technologies® (Coralville, IA). Complementary sequences were annealed and the resulting double-stranded DNA was ligated to Xho I/Not I-digested pLemiR™ vector (Open Biosystems®, Huntsville, AL). A549 cells were then infected with plasmids using the Trans-Lentiviral™ GIPZ packing system (Open Biosystems; Huntsville, AL) according to the manufacturer's protocol. Briefly, TLA-HEK293TTM cells were transfected using Arrest-In™ with 37.5 µg plasmid DNA in serum free medium for 4 hours. Media was then replaced with serum containing media for 36 hours. Media were collected, centrifuged to remove cell debris and used to transfect A549 and BEAS-2B cells. Forty eight hours after addition of virus, transfected cells were selected for by adding 2 µg/ml puromycin to growth medium.

### Real Time RT-PCR (qPCR) for Quantification of Small RNAs

Total RNA (20 ng), isolated from cells using PureLink™ Micro-to-Midi total RNA isolation kit (Invitrogen) according to the manufacturer's protocol, was reverse transcribed using TaqMan™ reverse transcription kit (Applied Biosystems®, Foster City, CA) and RNA-specific primers provided with TaqMan™ microRNA assays (Applied Biosystems®) in 15 µl, with annealing at 16°C for 30 min followed by extension at 42°C for 30 min. 1.33 µL of the RT reaction was then used with 1 µL specific primers for either *RNU6B*, *miR-30a*, or *miR-191* (Applied Biosystems®, Foster City, CA) in triplicate wells for 44-cycle PCR on a 7900HT thermocycler (Applied Biosystems®). The denaturation step at 95°C was for 15 s, and the annealing and extension step at 60°C was for 1 min. SDS software (Applied Biosystems®, Foster City, CA) was used to determine cycle-threshold (Ct) values of the fluorescence measured during PCR. Prism 5.0b software (GraphPad®; La Jolla, CA) was used for statistical analysis and graphing.

### Pathway Enrichment Analysis

The publicly available miRgator software (http://genome.ewha.ac.kr/miRGator/miRGator.html) was used to perform pathway enrichment analysis. The specific miRNAs selected were *miR-191* and -*30a-5p*. The target selection algorithm chosen was miRanda [3].

### Cell Proliferation Assay

Cell proliferation assays were performed using the Cell Titer 96® kit (Promega; Madison, WI) which uses the conversion of MTS (3-(4,5-dimethylthiazol-2-yl)-5-(3-carboxymethoxyphenyl)-2-(4-sulfophenyl)-2H-tetrazolium, inner salt) to formazan to measure cell viability. Briefly, cells were plated on 96 well plates at 4,000 cells in 100 µL per well. Cells were harvested in quadruplicate at various time points for 150 hours. To the harvested cells, 20 µL of assay reagent was added and viable cells were measured by absorbance at 450 nm 1 hour later on a Multiskan Plus (MTX Lab Systems; Vienna, VA) plate reader. Absorbance values were normalized to media control.

### Adherent Colony Formation

Cells in culture were trypsinized and plated in duplicate in 6-well plates at 200 and cells per well for A549 and 500 cells per well for BEAS-2B cell line derivatives respectively. Cells were allowed to grow at 37°C, 5% CO2 with media changes every 3–4 days until colonies were visible by eye. Media was aspirated and cells were stained with 0.2% methylene blue in 40% methanol for 30 minutes. Cells were washed and plates were scanned using an Epson Perfection V700 Photo scanner as a transparency. Quantitation of colonies was done using ImageJ software (Research Services Branch, National Institute of Mental Health, Bethesda).

### Soft Agar Colony Formation Assay

Base agar/media mix was added to 6-well plates (final concentration of 0.7% agar, 1x +DMEM, 10% FBS, 2 µg/ml puromycin) to coat the plate and allowed to polymerize. Cells suspensions were made to 100 cells/ml in media/agar mix (final concentration of 0.35% agar, 1x +DMEM, 10% FBS, 2 µg/ml puromycin). 1.5 ml of the cell suspension was plated on top of the base agar in each well and allowed to polymerize. 2 ml complete media was added on top of the agar once polymerized. The plates were put in 37°C, 5% CO2, and overlay media was changed every 3–4 days. Cultures were grown until a few colonies were visible by eye, 2 mg/ml Thiazolyl Blue Tetrazolium Bromide in PBS was added to the cells and were returned to the incubator until colonies were stained. Once colonies were stained blue, the stain was removed from the wells and pictures were taken using Cannon Powershot A590IS camera.

### In Vitro Scratch Assay

To estimate differences in cell migration, an in-vitro scratch assay was performed. Cross-hairs were drawn on the bottom of 6-well plates for orientation for imaging. Cells were plated in triplicate wells of at high density to reach 100% confluence 24 hours later. A scratch was then made directly next to the vertical line drawn on the plate using moderate and consistent pressure using a 10 µL tip and the plate lid as a straight edge. Scratches were imaged immediately on an Axio Observer microscope (Zeiss; Maple Grove, MN) at 5X magnification and orientation to the cross-hairs noted. Cells were then returned to 37°C, 5% CO2 and imaged at various time points until all scratches were closed.

### Xenograft Formation Assay

All in vivo studies were approved by the Institutional Animal Care and Use Committee of the Roswell Park Cancer Institute. Control and over-expressing A549 cell lines (6.0×105 cells) were injected subcutaneously between the shoulder blades of 4–5 week old SCID mice (5 mice in each cohort). Tumors were monitored 2–3 times per week by caliper measurements until the largest tumor was 2 cm in longest direction, at which point all mice were sacrificed. Tumors were dissected and weighed.

### Cell Cycle Analysis

Cells grown to 70%–90% confluence were detached by trypsinization, fixed in 70% ethanol at 4° C for 1–2 days, washed with PBS, and incubated at a density of 1−2×10^6^ cells/ml with 0.3 µM 4,6-diamidino-2-phenylindole dihydrochloride (DAPI; MP Biochemicals®, Solon, OH) in PBS at room temperature in the dark for 100 min. After washing once with PBS, DAPI fluorescence from cells was assayed by flow cytometry using an LSR II (BD Biosciences®, San Jose, CA) instrument equipped with a 408 nm violet laser diode and a 450/50 nm emission filter.

### Drug Sensitivity Assay

After cells were grown to 50%–60% confluence in 96-well plates (5 wells per cell-line), the medium was replaced with that containing 1 µg/ml doxorubicin hydrochloride (Enzo Life Sciences®, Farmingdale, NY) or cisplatin (Calbiochem®, San Diego, CA). After 1.5–2 days of culture, viability of cells was measured as described for the cell proliferation assay and compared to those that were not exposed to the drugs.

## Results

### Creation of Stable Cell Lines with Over-Expression of miR-30a and -191

**Figure 1:**
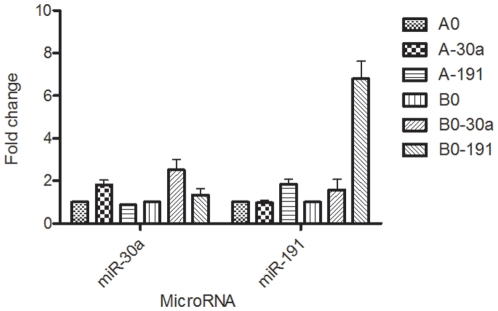
Quantitative RT-PCR for confirming over-expression of *miR-30a* in A-30a and B-30a, and of *miR-191* in A-191 and B-191 cell lines, respectively. Expression is normalized to control cell lines (A0 or B0).

Stable cell lines overexpressing *miR-30a* and *-191* were generated using a lentiviral system as described above. *MiR-30a* was overexpressed ∼2 fold and ∼3 fold in both A549 (A-30a) and BEAS-2B (B-30a) respectively compared to controls (A0 and B0 respectively). Compared to controls, *miR-191* was overexpressed ∼2 fold and ∼7 fold in A549 (A-191) and BEAS-2B (B-191) respectively (Figure 1). Overexpression was also confirmed by visualization of RFP containing cells by microscopy.

### Pathway Enrichment Analysis

Target prediction by the miRanda algorithm identified 731 targets for *miR-30a-5p* and 570 targets for *miR-191*. The top ten pathways identified by the pathway enrichment analysis included pathways influencing cell cycle, cell proliferation, drug resistance and cell motility, among others (Table 1).

**Table 1:** Top ten pathways identified by pathway enrichment analysis for *miR-30a* and *-191*.

Pathway database	Description of pathway	P
*miR -191*		
BioCarta	CDK Regulation of DNA Replication	0.0001
GenMAPP	Cell Cycle	0.0011
BioCarta	Role of MEF2D in T-cell Apoptosis	0.0013
BioCarta	Y branching of actin filaments	0.0015
BioCarta	Phospholipase C d1 in phospholipid associated cell signaling	0.0019
BioCarta	Oxidative Stress Induced Gene Expression Via Nrf2	0.0024
BioCarta	Nitric Oxide Signaling Pathway	0.0028
BioCarta	Stathmin and breast cancer resistance to antimicrotubule agents	0.0036
BioCarta	Cell Cycle: G2/M Checkpoint	0.0041
BioCarta	Control of Gene Expression by Vitamin D Receptor	0.0041
*miR-30a*		
BioCarta	Cyclins and Cell Cycle Regulation	0.0013
BioCarta	Classical Complement Pathway	0.003
BioCarta	ChREBP regulation by carbohydrates and cAMP	0.0094
BioCarta	Adhesion and Diapedesis of Granulocytes	0.0107
BioCarta	Complement Pathway	0.0107
BioCarta	Neutrophil and Its Surface Molecules	0.0119
BioCarta	Alternative Complement Pathway	0.0149
BioCarta	Cyclin E Destruction Pathway	0.0183
BioCarta	Cell Cycle: G1/S Check Point	0.0218
BioCarta	Ghrelin: Regulation of Food Intake and Energy Homeostasis	0.0218
BioCarta	Monocyte and its Surface Molecules	0.0218

### Over-Expression of *miR-30a* or *-191* Does Not Alter Measures of Proliferation Both In Vitro and In Vivo in A-549 and BEAS-2B Cell Lines

**Figure 2:**
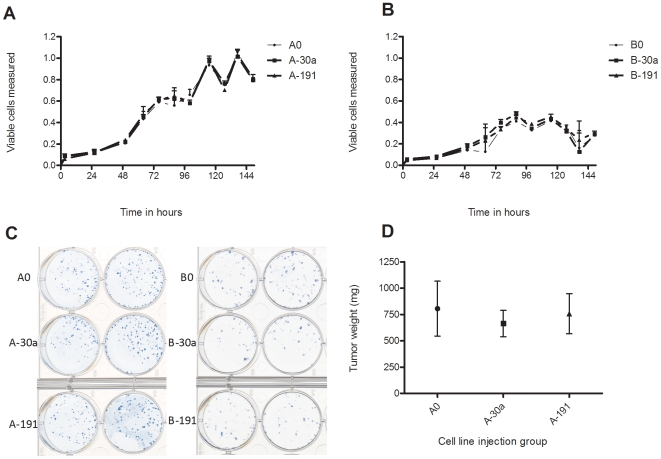
Overexpression of miR-30a and -191 does not alter A549 and BEAS-2B cellular proliferation in-vitro and in-vivo. A,B. Cell proliferation curves for cell lines A0, A-30a and A-191 (A) and B0, B-30a and B-191(B) demonstrating no change in cell proliferation. C. Methylene blue stained cell culture plates demonstrating no difference in adherent colony formation in A-30a and A-191 compared to A0 and in B-30a and B-191 compared to B0. D. Xenograft formation in SCID mice by A549 is not altered by over-expression of *miR-30a* or *-191*.

Pathway enrichment analysis suggested that *miR-30a* and *-191* influenced cell cycle and cell proliferation. To examine the effect of overexpression of *miR-30a* and *-191* on A549 and BEAS-2B, growth curves were plotted based on measurements of cell viability at different time points over a 150-hour period. No difference in growth curves were seen in A-30a and A-191 compared to A0 and in B-30a and B-191 compared to B0 (Figure 2A, 2B). Cell proliferation in different cell lines was also compared using adherent colony formation. No difference was seen in either number or size of adherent colonies in cell lines over-expressing *miR-30a* or *-191* compared to the corresponding control cell line (Figure 2C).

Anchorage independence is one of the hallmarks of transformation. We sought to determine the effect of overexpression of *miR-30a* or *-191* on anchorage independent growth of A549 and BEAS-2B cells using the soft agar colony assay as described above. All lines formed very few colonies that were visible by microscopy with only 2–3 colonies visible by naked eye. No differences were seen after staining or by microscopy (data not shown).

Xenograft formation is thought to represent a biologically relevant measure of tumor aggressiveness. We assessed the effect of overexpression of *miR-30a* or *-191* on xenograft formation in SCID mice by A549 cells. When compared to the control cell line (A0), A-30a and A-191 do not form larger xenografts (Figure 2D). The increase of tumor volume over time is also no different in these cell lines compared to control (data not shown). As the BEAS-2B cell line is non-tumorigenic, we did not assess xenograft formation using B0, B-30a or B-191.

### The Effect of Over-Expression of *miR-30a* or *-191* on Cell Migration and Cell Cycle Distribution of A-549 and BEAS-2B Cell Lines

**Figure 3:**
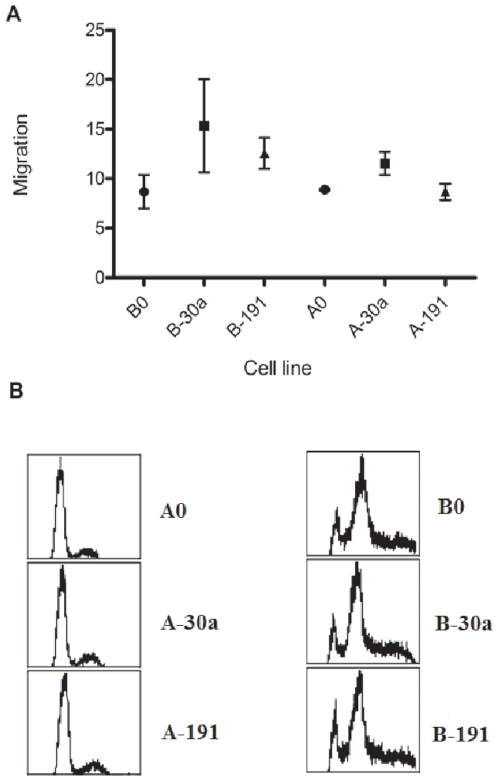
Analysis of migration and cell cycle with overexpression of miR-30a and -191. A. Cell migration of A-30a and B-30a is increased compared to controls; no effect of overexpression of *miR-191* is seen. B. Cell cycle analysis of stable transfectants demonstrating no difference between cells over-expressing *miR-30a* and *-191* compared to controls.

Increased cell migration is an important mechanism that is thought to increase metastatic potential of cancer cells. This may be independent of cell proliferation rates. Therefore, we studied the effect of *miR-30a* or *-191* on cell migration of A549 and BEAS-2B cell lines using the scratch assay as described above. There is a modest increase in the migration of A-30a and B-30a compared to A0 and B0 respectively. There was no significant difference in the migration distance between cell lines overexpressing *miR-191* when compared to the corresponding control cell lines (Figure 3A).

An examination of the predicted targets of *miR-30a* and *-191* and the cellular processes influenced by them shows a statistically significant enrichment of pathways involving the cell cycle. Therefore, we studied the effect of overexpression of *miR-30a* or *-191* on cell cycle distribution. Flow cytometry after staining with DAPI was used to perform cell cycle analysis of all cell lines. Compared to the controls, cell lines over-expressing *miR-30a* or *-191* do not show significant changes in the cell cycle distribution (Figure 3B).

### Over-Expression of *miR-30a* or *-191* Does Not Alter Chemosensitivity to Doxorubicin or Cisplatin

**Figure 4:**
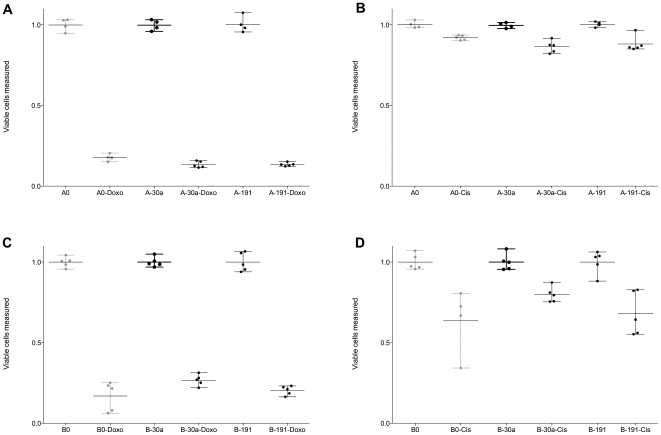
Chemosensitivity is not altered by over-expression of *miR-30a* or *-191.* Sensitivity of A549 cells (A,B) and BEAS-2B cells (C,D) to doxorubicin and cisplatin. Grey indicates control cells.

Doxorubicin and cisplatin are commonly used chemotherapeutic agents. A549 is moderately sensitive to cisplatin and doxorubicin (http://dtp.nci.nih.gov), enabling the assessment of both increased and decreased sensitivity to these drugs. Overexpression of *miR-30a* or *-191* does not alter the chemosensitivity of A549 and BEAS-2B to either drug (Figure 4).

## Discussion

MiRNA changes are commonly seen in cancerous tissue compared to their normal counterparts. The magnitude of these changes are modest and their functional significance in unknown. One of the difficulties in the study of miRNAs are that one miRNA has several hundred predicted targets and these bio-informatic predictions are not very accurate. Also, the role of miRNAs in a cell depends on the genetic constitution and the transcriptome of a cell at any given time and this complexity is not captured simply by their forced over-expression. However, phenotypic alterations secondary to forced over-expression can provide clues to understand their contribution to the malignant phenotype. In this study, we generated stable transfectants that constitutively over-express *miR-30a* or *-191* and do not demonstrate significant changes in phenotype in the various assays that were performed. A number of possible explanations can explain this lack of alteration of phenotype. First, it is possible that the level of expression of the miRNAs achieved is not enough to create a difference. Even though the fold changes seen by microarray experiments is typically modest, such experiments may underestimate actual changes in malignant epithelial cells due to the dilution of changes in malignant epithelial cells by stromal cells that constitute a significant proportion of pathological specimens. Also, the magnitude of changes seen by microarrays is often smaller than the corresponding changes seen by RT-PCR. Therefore, the actual changes in malignant cells may be more than that achieved in our model system. Conversely, it is possible that the changes seen in miRNA microarrays spuriously overestimate changes. For example, in a study by Peltier et al[18] that carefully evaluated several candidate miRNAs for stability of expression across five types of cancer and normal tissues by RT-PCR, *miR-191* was so stably expressed that it was proposed as a gene to be used for normalization. This argues against a role for *miR-191* in carcinogenesis. Therefore, selecting miRNAs based on microarray data may not be a good strategy. Third, all miRNAs may not be able to result in phenotypic changes on their own; their effect may be context specific and may need to be coupled with alterations of expression of either other miRNAs or mRNAs to alter phenotype.

In summary, we conclude that over-expression of *miR-30a* or *-191* does not lead to an alteration in cell cycle, proliferation, xenograft formation and chemosensitivity of A549 and BEAS-2B cell lines. Using microarray data from whole tumors to select specific miRNAs for functional study may be a sub-optimal strategy. It is important to confirm the functional significance of changes seen in miRNA expression data in specific tumor types to define the biological roles of specific miRNAs.

## References

[1] Lee RC, Feinbaum RL, Ambros V (1993). The C. elegans heterochronic gene lin-4 encodes small RNAs with antisense complementarity to lin-14.. Cell.

[2] Lewis BP, Burge CB, Bartel DP (2005). Conserved seed pairing, often flanked by adenosines, indicates that thousands of human genes are microRNA targets.. Cell.

[3] John B, Enright AJ, Aravin A, Tuschl T, Sander C (2004). Human MicroRNA targets.. PLoS Biol.

[4] Yanaihara N, Caplen N, Bowman E, Seike M, Kumamoto K (2006). Unique microRNA molecular profiles in lung cancer diagnosis and prognosis.. Cancer Cell.

[5] Rodriguez A, Griffiths-Jones S, Ashurst JL, Bradley A (2004). Identification of mammalian microRNA host genes and transcription units.. Genome Res.

[6] Calin GA, Liu CG, Sevignani C, Ferracin M, Felli N (2004). MicroRNA profiling reveals distinct signatures in B cell chronic lymphocytic leukemias.. Proc Natl Acad Sci U S A.

[7] Volinia S, Calin GA, Liu CG, Ambs S, Cimmino A (2006). A microRNA expression signature of human solid tumors defines cancer gene targets.. Proc Natl Acad Sci U S A.

[8] Schetter AJ, Leung SY, Sohn JJ, Zanetti KA, Bowman ED (2008). MicroRNA expression profiles associated with prognosis and therapeutic outcome in colon adenocarcinoma.. Jama.

[9] Szafranska AE, Davison TS, John J, Cannon T, Sipos B (2007). MicroRNA expression alterations are linked to tumorigenesis and non-neoplastic processes in pancreatic ductal adenocarcinoma.. Oncogene.

[10] Budhu A, Jia HL, Forgues M, Liu CG, Goldstein D (2008). Identification of metastasis-related microRNAs in hepatocellular carcinoma.. Hepatology.

[11] Garzon R, Volinia S, Liu CG, Fernandez-Cymering C, Palumbo T (2008). MicroRNA signatures associated with cytogenetics and prognosis in acute myeloid leukemia.. Blood.

[12] Mellios N, Huang HS, Grigorenko A, Rogaev E, Akbarian S (2008). A set of differentially expressed miRNAs, including miR-30a-5p, act as post-transcriptional inhibitors of BDNF in prefrontal cortex.. Hum Mol Genet.

[13] Hand NJ, Master ZR, Eauclaire SF, Weinblatt DE, Matthews RP (2009). The microRNA-30 family is required for vertebrate hepatobiliary development.. Gastroenterology.

[14] Roldo C, Missiaglia E, Hagan JP, Falconi M, Capelli P (2006). MicroRNA expression abnormalities in pancreatic endocrine and acinar tumors are associated with distinctive pathologic features and clinical behavior.. J Clin Oncol.

[15] Izzotti A, Calin GA, Arrigo P, Steele VE, Croce CM (2009). Downregulation of microRNA expression in the lungs of rats exposed to cigarette smoke.. Faseb J.

[16] Giard DJ, Aaronson SA, Todaro GJ, Arnstein P, Kersey JH (1973). In vitro cultivation of human tumors: establishment of cell lines derived from a series of solid tumors.. J Natl Cancer Inst.

[17] Ke Y, Reddel RR, Gerwin BI, Reddel HK, Somers AN (1989). Establishment of a human in vitro mesothelial cell model system for investigating mechanisms of asbestos-induced mesothelioma.. Am J Pathol.

[18] Peltier HJ, Latham GJ (2008). Normalization of microRNA expression levels in quantitative RT-PCR assays: identification of suitable reference RNA targets in normal and cancerous human solid tissues.. Rna.

